# Analysis of the effects of perfluorooctane sulfonate on COPD using network toxicology and molecular docking

**DOI:** 10.1016/j.isci.2025.113372

**Published:** 2025-08-18

**Authors:** Chenwen Peng, Jingyan Wei, Wuxia Chen, Fengao Liang, Zhenyan Huang, Ying Huang

**Affiliations:** 1 Zhongshan Hospital of Traditional Chinese Medicine Affiliated to Guangzhou University of Traditional Chinese Medicine, Zhongshan 528400, China; 2 Zhongshan Hospital of Traditional Chinese Medicine, Zhongshan 528400, China

**Keywords:** Applied computing, Chemistry, Environmental science

## Abstract

This study elucidates the pathogenic mechanisms of perfluorooctane sulfonate (PFOS) in chronic obstructive pulmonary disease (COPD) through network toxicology and molecular docking. By integrating multiple databases, we identified 158 PFOS-related targets in COPD, with five key proteins (epidermal growth factor receptor [EGFR], ESR1, GRB2, HSP90AA1, and SRC) showing central roles in protein interaction networks. Functional enrichment analysis revealed their involvement in key pathophysiological processes, including airway inflammatory responses, oxidative stress, and immune regulation, primarily through modulation of cell survival and proliferation pathways and immune and hormonal regulation pathways. Gene set enrichment analysis (GSEA) further validated these findings by confirming the significant enrichment of five key KEGG pathways identified in our analysis. Molecular docking studies confirmed high-affinity binding between PFOS and these core targets, indicating PFOS may dysregulate inflammatory responses, oxidative balance, and cellular proliferation in COPD pathogenesis. These findings provide critical molecular insights into environmental pollutant-aggravated respiratory disorders and highlight potential intervention targets for COPD management.

## Highlights

• Network toxicology and molecular docking elucidated PFOS toxicity mechanisms
• GSEA, GO, and KEGG revealed key pathways in PFOS-induced COPD
• Key targets for PFOS-induced COPD: EGFR, ESR1, GRB2, HSP90AA1, and SRC

## Introduction

Perfluorooctane sulfonate (PFOS) is a synthetic fluorinated organic compound widely utilized in industrial, agricultural, and consumer applications, including textiles, furniture, food packaging, and electrical appliances, due to its exceptional chemical and thermal stability.^1^^,^^2^ However, its environmental persistence and bioaccumulative properties have raised significant concerns. Owing to its high resistance to hydrolysis, photolysis, and microbial degradation,^3^ PFOS is ubiquitously distributed in air, water, soil, and biota, with detections even in remote regions such as the Arctic and Antarctic.^4^ This widespread presence indicates that human exposure to PFOS in daily life is virtually unavoidable. Human exposure primarily occurs through the inhalation of dust, dermal contact, and the ingestion of contaminated food and water. Upon entering the body, PFOS binds to serum proteins and distributes to multiple organs, including the lungs, liver, and kidneys.^5^ With an elimination half-life of approximately 5 years in humans, PFOS accumulates in these organs over extended periods, leading to potential toxic effects.^6^ Unlike traditional toxicants, which typically target single proteins or genes, PFOS disrupts complex protein and gene networks, resulting in systemic effects that are challenging to predict and mitigate.^7^ The strong resistance of PFOS to degradation and its long-term bioaccumulation in both the environment and human tissues underscore the necessity for comprehensive health risk assessments and a deeper understanding of its toxicological mechanisms. Elucidating the multifaceted interactions of PFOS with biological systems is critical for developing effective strategies to minimize its adverse impacts on human health and the environment.

Exposure to PFOS can impact pulmonary health through multiple biological pathways, potentially increasing the risk of developing COPD. The NHANES-based epidemiological study demonstrated significantly elevated PFOS levels in COPD patients, with sex-specific associations suggesting PFOS may contribute to COPD pathogenesis particularly in males.^8^ Another study found that serum PFOS exposure significantly increased COPD risk, particularly in older adults and smokers, while moderate physical activity mitigated this effect.^9^ These findings support the biological plausibility of PFOS contributing to COPD pathogenesis through environmental exposure pathways. Research has shown that PFOS exposure promotes reactive oxygen species (ROS) production, leading to oxidative stress and compromising the repair ability of airway epithelial cells, thereby exacerbating lung injury and promoting the onset of respiratory diseases.^10^ Furthermore, PFOS exposure has been shown to suppress immune function, characterized by reduced antibody production, decreased spleen and thymus weights, and a decline in cell counts, ultimately impairing the body’s ability to clear viruses and bacteria and increasing the risk of respiratory infections.^11^^,^^12^^,^^13^ Further research has revealed that PFOS may exacerbate airway inflammation and pulmonary tissue remodeling by triggering pro-inflammatory signaling cascades, including nuclear factor κB (NF-κB), which results in the secretion of inflammatory mediators like interleukin-6 (IL-6) and tumor necrosis factor alpha (TNF-α).^14^ These pathological alterations are hallmark features of COPD. Despite notable advancements in elucidating the involvement of PFOS in COPD pathogenesis, its precise molecular mechanisms remain incompletely elucidated. Therefore, comprehensive and innovative research approaches are essential to effectively assess the health risks associated with PFOS contamination and to further elucidate its underlying toxicological mechanisms.

This study aims to utilize network toxicology approaches to pinpoint crucial molecular targets and pivotal signaling cascades implicated in PFOS-induced COPD and to validate the binding relationships between PFOS and its prospective targets through molecular docking techniques, thereby elucidating its toxicological mechanisms. By integrating network toxicology and molecular docking techniques, we can systematically identify key molecular targets and associated biological pathways of PFOS, thereby comprehensively elucidating its potential impact on COPD. This innovative approach not only offers an efficient methodology for evaluating the role of PFOS in COPD but also provides critical scientific foundations for developing therapeutic interventions and risk assessment strategies targeting PFOS-related diseases.

## Results

### Toxicity assessment of PFOS

**Figure 1:**
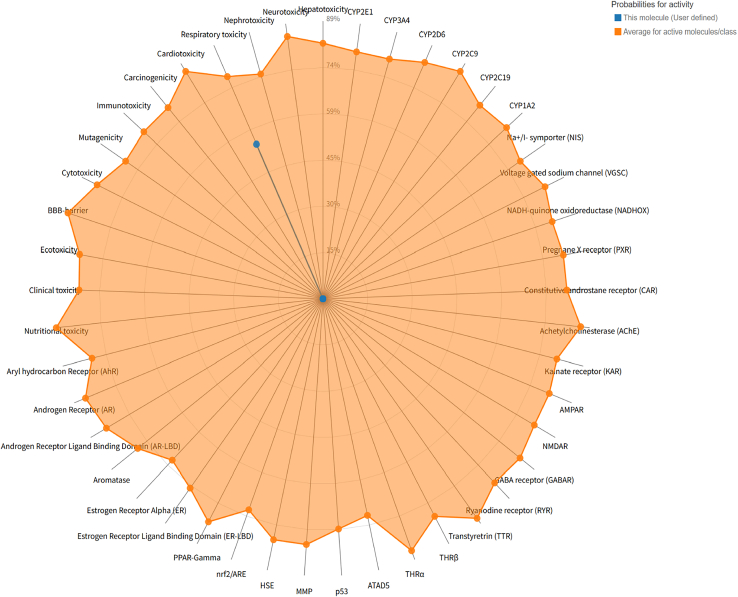
Radar chart of the toxicity of PFOS

By integrating the toxicity analysis results from ProTox-II and ADMETlab, we generated a radar chart illustrating the toxicity profile of PFOS (Figure 1; Tables 1 and 2). The analysis consistently indicated that the active toxicity endpoints of PFOS are closely associated with respiratory toxicity.

**Table 1:** Toxicity model report for ProTox-3.0

Classification	Target	Shorthand	Prediction	Probability
Organ toxicity	Hepatotoxicity	Dili	inactive	0.89
Organ toxicity	Neurotoxicity	Neuro	inactive	0.94
Organ toxicity	Nephrotoxicity	Nephro	inactive	0.52
Organ toxicity	Respiratory toxicity	Respi	active	0.54
Organ toxicity	Cardiotoxicity	Cardio	inactive	0.88

**Table 2:** Toxicity model report for ADMETlab 3.0

Property	Value	Comment
Respiratory	0.805	Category 1: respiratory toxicants; category 0: non-respiratory toxicants. The output value is the probability of being toxic, within the range of 0–1

### Identification of PFOS-induced COPD targets

**Figure 2:**
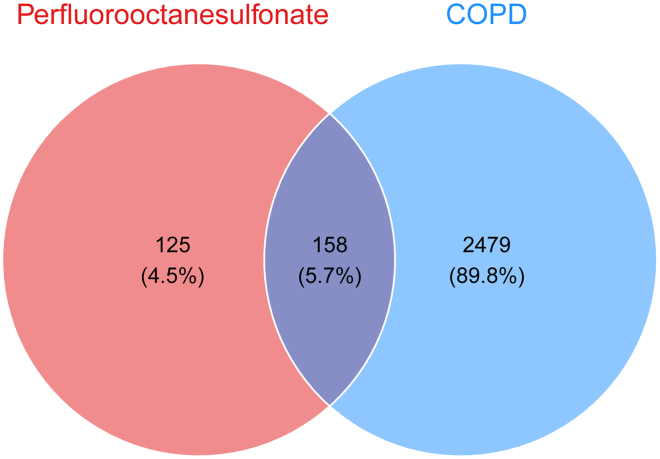
Venn diagram of potential targets of PFOS and COPD

We initially screened 283 potential targets associated with PFOS from the STITCH database and the Swiss Target Prediction database. Subsequently, by consolidating information sourced from the GeneCards, OMIM, and TTD databases, we identified 2,637 targets highly associated with COPD. To further elucidate the molecular connections between PFOS and COPD, we performed an intersection analysis of the two target groups utilizing the Venny 2.1.0 online tool, ultimately identifying 158 shared targets between PFOS and COPD (Figure 2). These intersecting targets are considered potential key molecules in PFOS-induced COPD.

### PPI network construction and core target screening

**Figure 3:**
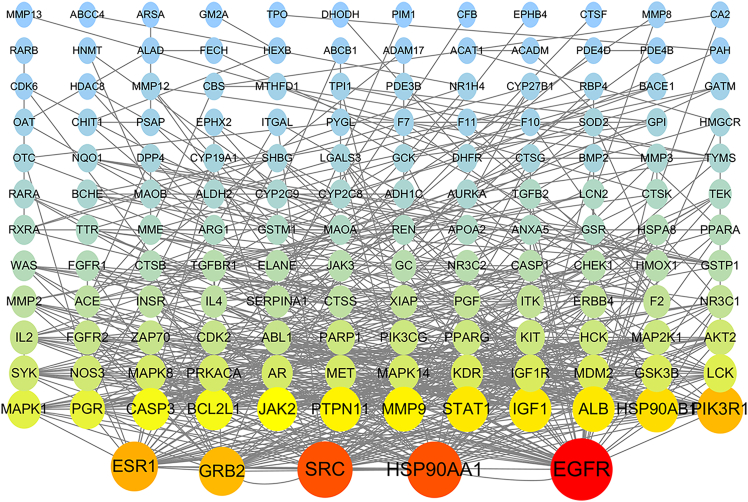
The PPI network of the potential action targets of PFOS-induced COPD

Employing the STRING database, we generated a protein-protein interaction (PPI) network consisting of 158 nodes and 1,989 edges, exhibiting an average node degree of 25.2, indicating extensive interactions among the targets. Subsequently, topological analysis of the network was conducted utilizing Cytoscape software (version 3.10.1), generating an intuitive visualization of the PPI network structure (Figure 3). In this visualization, the color depth and size of the nodes are proportional to their importance within the network, with darker colors and larger areas indicating higher centrality and greater criticality in the network. Based on the analysis of topological parameters such as degree centrality, betweenness centrality, and closeness centrality, we ultimately identified five core targets, including epidermal growth factor receptor (EGFR), ESR1, GRB2, HSP90AA1, and SRC. These targets exhibit significant connectivity and functional importance within the network and are considered key molecules in PFOS-induced COPD.

### Functional and pathway enrichment analysis of core targets

**Figure 4:**
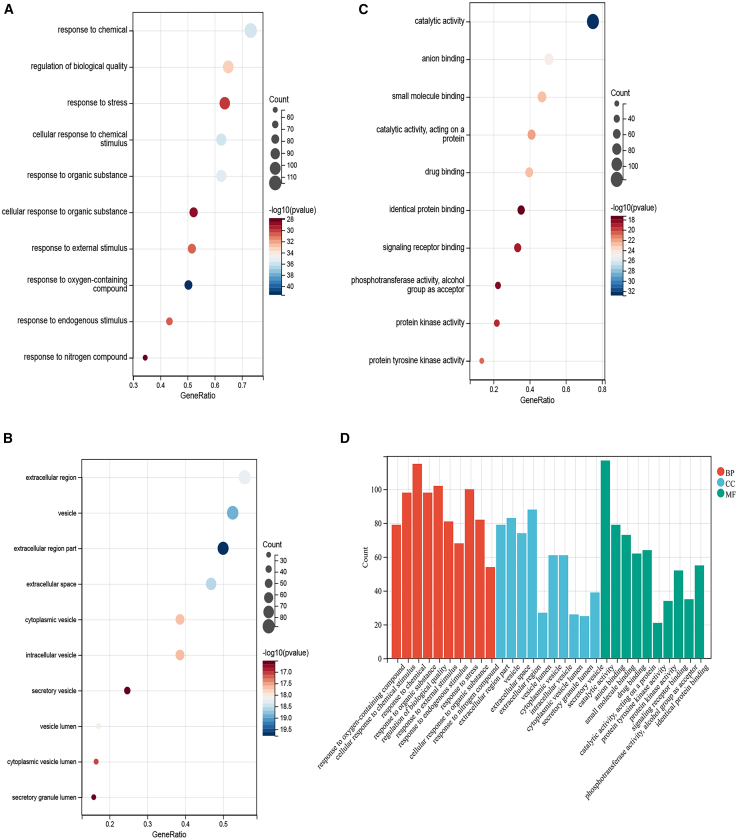
GO enrichment analysis of the core genes of PFOS and COPD (A–C) Biological processes (BP; A), cellular components (CC; B), and molecular functions (MF; C). The size of each bubble indicates the number of genes involved in the pathway, with cooler blue colors representing greater enrichment significance. (D) The histogram illustrates the top 10 enriched terms for each GO category (BP, CC, and MF). The most significant pathways are positioned on the far left for each category, with significance decreasing progressively toward the right.

**Figure 5:**
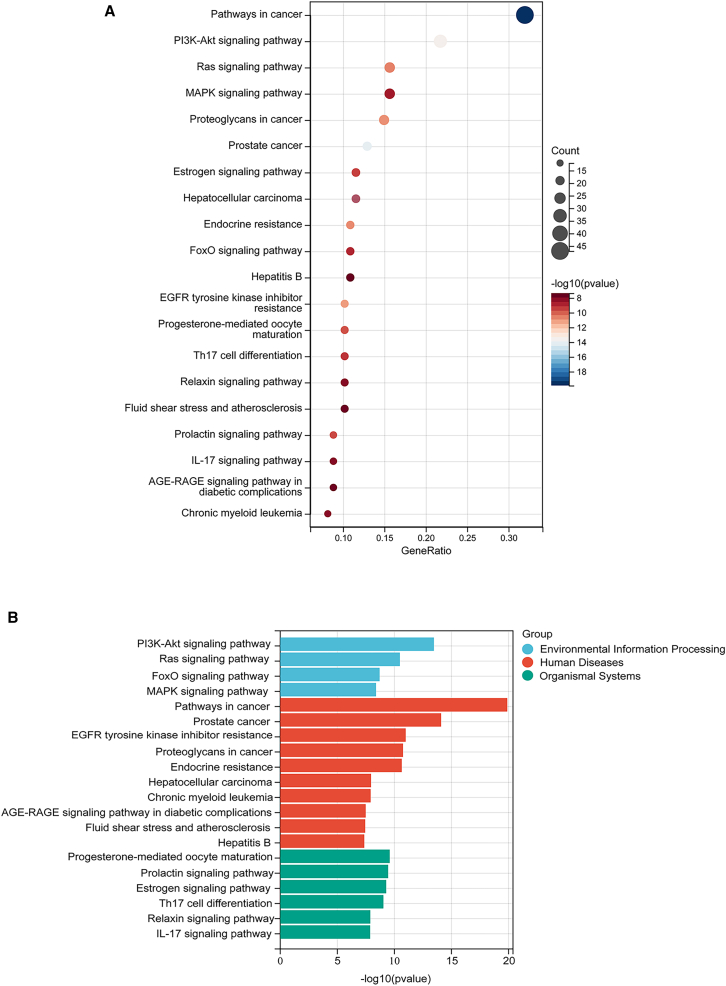
KEGG enrichment analysis of the core genes of PFOS and COPD (A) In the bubble plot (A), the size of each bubble corresponds to the number of genes involved in a specific pathway. The cooler the color of the bubble, the more significant the enrichment. (B) In the histogram (B), the length of each bar represents the *p* value, with longer bars indicating higher significance.

The analysis results identified 3,547 significantly enriched Gene Ontology (GO) terms, systematically categorized into 3,059 biological processes (BPs), 140 cellular components (CCs), and 348 molecular functions (MFs). To identify the most representative GO terms, we ranked the results based on *p* values and selected the top 10 entries with the lowest *p* values from each category (BPs, CCs, and MFs), as shown in Figure 4. The results revealed that the biological processes primarily involved response to chemical, regulation of biological quality, and cellular response to chemical stimulus; cell components encompassed extracellular region, vesicle, and extracellular space; and the molecular functions might be related to catalytic activity, anion binding, and small molecule binding. Additionally, we performed KEGG pathway enrichment analysis on the 158 potential targets using the SangerBox platform. The analysis identified a total of 157 significantly enriched signaling pathways, and the results were visualized using a statistical significance bubble plot and a categorical histogram (Figure 5). These graphical representations displayed the 20 most significant enriched KEGG pathways, ranked in descending order based on their *p* values. They provided an intuitive representation of the pathways most pertinent to this investigation and highlighted their importance. KEGG pathway analysis revealed significant enrichment in multiple signaling cascades, notably cancer-related pathways, PI3K-Akt, mitogen-activated protein kinase (MAPK), Ras, estrogen, and IL-17 associated with diabetic complications.

### Results of GSEA

**Figure 6:**
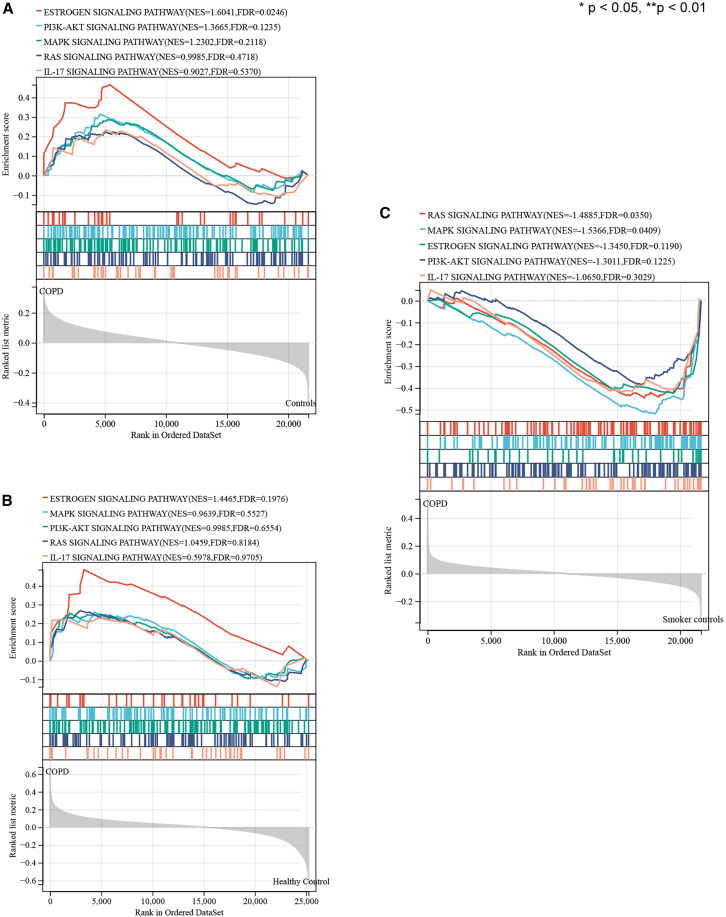
Gene set enrichment analysis (GSEA) of KEGG pathways in different datasets (A–C) GSEA identifies significant KEGG signaling pathways in PFOS-induced COPD across datasets (A: GSE54837; B: GSE76925; C: GSE130928). Significance thresholds: |NES| >1, nominal ∗*p* < 0.05, ∗∗*p* < 0.01, FDR q < 0.25 (weighted statistics, 1,000 permutations). ES, enrichment score; NP, nominal *p* value.

In order to understand whether the KEGG pathway was statistically significant in the test group and the control group, “PI3K-Akt signaling pathway,” “Ras signaling pathway,” “estrogen signaling pathway,” “MAPK signaling pathway,” and “IL-17 signaling pathway” were used to conduct GSEA. Pathways meeting the condition of |NES| >1, *p* value < 0.05, and FDR<0.25 were considered statistically significant. The GSEA results were shown in Table 3 and Figure 6. In GSE5483, the pathway that met the conditions was estrogen signaling pathway (|NES| = 1.6041, ES = 0.4657, *p* value = 0.0074, FDR = 0.0246). Notably, while the PI3K-Akt pathway enrichment failed to meet significance thresholds (|NES| = 1.3665, ES = 0.3146, *p* value = 0.0754, FDR = 0.1235), its enrichment trend suggests potential biological relevance worthy of further study. There was no pathway that met the inclusion criteria in GSE76925, while the estrogen signaling pathway was close to the saliency condition (|NES| = 1.4465, ES = 0.4864, *p* value = 0.0558, FDR = 0.1976). Ras signaling pathway (|NES| = 1.4885, ES = −0.4528, *p* value = 0.0201, FDR = 0.0350) and MAPK signaling pathway (|NES| = 1.5366, ES = −0.5268, *p* value = 0.0188, FDR = 0.0409) were screened in GSE130928. Notably, the identified pathways align with established molecular mechanisms underlying COPD pathogenesis, suggesting PFOS may exacerbate disease progression through these conserved signaling networks.

**Table 3:** GSEA results of KEGG pathways in three datasets

Datasets	KEGG pathways	ES	NES	*p* value	FDR
GSE54837	PI3K-Akt signaling pathway	0.3146	1.3665	0.0754	0.1235
	Ras signaling pathway	0.2248	0.9985	0.4542	0.4718
	Estrogen signaling pathway	0.4657	1.6041	0.0074	0.0246
	MAPK signaling pathway	0.2863	1.2302	0.1606	0.2118
	IL-17 signaling pathway	0.2331	0.9027	0.5945	0.5370
GSE76925	PI3K-Akt signaling pathway	0.2548	0.9985	0.4650	0.6554
	Ras signaling pathway	0.2673	1.0459	0.3740	0.8184
	Estrogen signaling pathway	0.4864	1.4465	0.0558	0.1976
	MAPK signaling pathway	0.2610	0.9639	0.5275	0.5527
	IL-17 signaling pathway	0.2283	0.5978	0.9499	0.9705
GSE130928	PI3K-Akt signaling pathway	−0.3930	−1.3011	0.1008	0.1225
	Ras signaling pathway	−0.4528	−1.4885	0.0201	0.0350
	Estrogen signaling pathway	−0.4646	−1.3450	0.1138	0.1190
	MAPK signaling pathway	−0.5268	−1.5366	0.0188	0.0409
	IL-17 signaling pathway	−0.4405	−1.0650	0.4056	0.3029

### Molecular docking validation of PFOS and COPD

**Figure 7:**
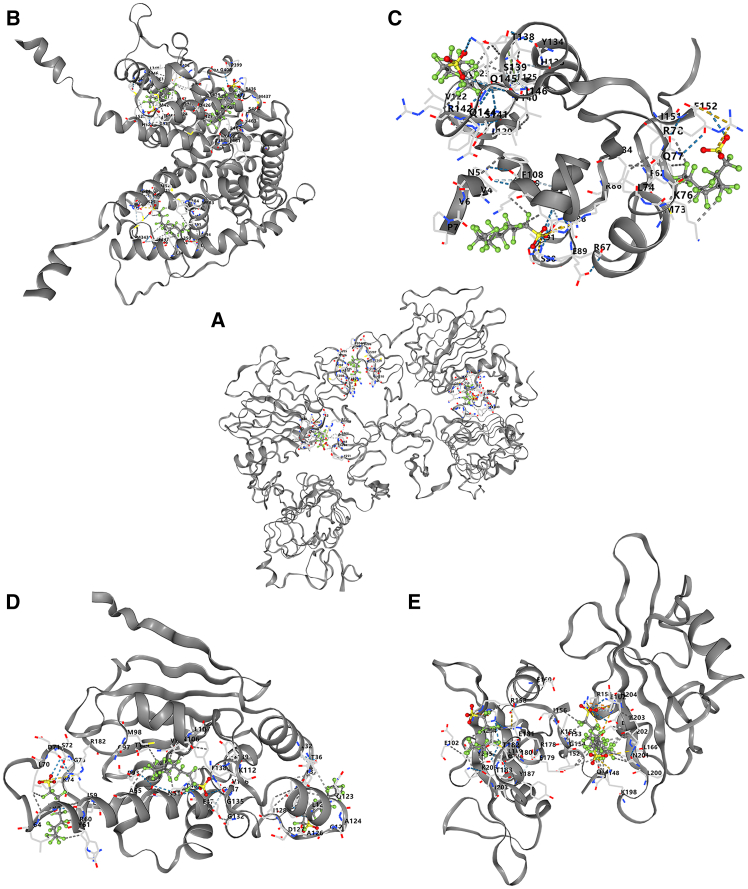
Molecular docking analysis of PFOS with core targets (A–E) PFOS and EGFR (A); PFOS and ESR1 (B); PFOS and GRB2 (C); PFOS and HSP90AA1 (D); PFOS and SRC (E).

Through molecular docking analysis, we investigated the interactions between PFOS and five key target genes: EGFR, ESR1, GRB2, HSP90AA1, and SRC (Figure 7). Molecular docking simulations conducted using the CB-Dock2 online tool revealed that PFOS exhibited binding energies below 0 kcal/mol, with the top three highest-affinity ligands for each target protein (Table 4). This outcome underscores the spontaneous and potent binding affinity of PFOS to these pivotal proteins, thereby corroborating its robust interaction within the molecular pathways associated with chronic obstructive pulmonary disease (COPD) and highlighting its potential critical role in the disease’s etiology.

**Table 4:** Binding free energies for five protein targets

Core targets	Binding free energy 1 (kcal/mol)	Binding free energy 2 (kcal/mol)	Binding free energy 3 (kcal/mol)
EGFR	−8.8	−7.8	−7.5
ESR1	−9.8	−9.4	−8.1
GRB2	−6.9	−6.8	−5.7
HSP90AA1	−7.8	−6.3	−6.3
SRC	−8.1	−7.9	−6.5

This table presents the three lowest binding free energy values for five distinct targets (EGFR, ESR1, GRB2, HSP90AA1, and SRC). Lower (more negative) values indicate stronger binding affinity between the ligand and the target.

## Discussion

We utilized a comprehensive array of databases to acquire and analyze relevant target information, including PubChem, ProTox-II, ADMETlab, STITCH, Swiss Target Prediction, PharmMapper, GeneCards, OMIM, and STRING. Using Cytoscape software, we visualized and performed topological analysis on the protein-protein interaction (PPI) network to identify core targets. Subsequently, GO functional annotation and KEGG pathway enrichment analysis of the core targets were conducted using the SangerBox platform, systematically revealing the potential toxicity mechanisms of PFOS and its association with COPD. Notably, our KEGG pathway analysis identified five significantly enriched pathways that were further validated by GSEA, demonstrating consistent involvement in PFOS-associated COPD pathogenesis. Finally, molecular docking studies were performed using the RCSB Protein DataBank and the CB-Dock2 online tool to validate the molecular interactions between PFOS and core target proteins. In summary, this study explores the molecular pathways involved in PFOS-triggered COPD.

Through comprehensive network analysis, we identified five critical molecular targets: EGFR, ESR1, GRB2, HSP90AA1, and SRC. PFOS may contribute to the pathogenesis and progression of COPD by interacting with these molecular targets and their associated signaling pathways. Chronic exposure to stimuli such as PFOS, smoking, air pollution, or pathogen infection induces IL-33 secretion by airway epithelial cells. It is readily oxidized to IL-33oxox, which then forms a complex by binding to the receptor for advanced glycation end products (RAGE) and the epidermal growth factor receptor (EGFR). The formation of this complex activates EGFR, leading to the phosphorylation of its tyrosine residues and subsequent activation of downstream signaling pathways, including STAT5, ERK1/2, JNK, and PI3K-Akt.^15^ The activation of these signaling pathways promotes fibroblast activation and collagen deposition, leading to airway wall thickening and fibrosis, thereby exacerbating irreversible airflow limitation and lung function decline. Notably, the activation of JNK and ERK1/2 also stimulates fibroblasts to release pro-inflammatory mediators, such as TNF-α and IL-6, further amplifying the inflammatory response.^16^ In addition, EGFR activation by its ligands promotes mucus secretion, exacerbating symptoms such as coughing and sputum production in patients.^17^^,^^18^ These pathological changes collectively contribute to the pathogenesis and exacerbation of COPD. Emerging evidence highlights the multifaceted therapeutic potential of EGFR inhibition in COPD through distinct yet interconnected mechanisms. First, studies demonstrate that pharmacological blockade of EGFR and its downstream pathways—particularly via the active compound combination ECC-BYF—effectively reduces airway epithelial permeability, thereby preserving barrier integrity.^19^ Specifically, EGFR inhibitors counteract cigarette-smoke-induced damage by preventing calpain-mediated degradation of tight junction proteins, which in turn diminishes susceptibility to inhaled pathogens.^20^ Beyond epithelial protection, preclinical murine models reveal that EGFR-targeted therapy promotes neutrophil apoptosis and attenuates neutrophil-driven pulmonary inflammation, suggesting broader anti-inflammatory benefits.^21^ Notably, these agents also address systemic manifestations of COPD, such as locomotor muscle dysfunction, by inducing a fiber-type shift toward slow-twitch oxidative phenotypes, thereby improving mitochondrial efficiency and exercise tolerance.^22^ Finally, EGFR inhibition may alleviate mucus hypersecretion by suppressing mechanical-stress-induced ERK/MUC5AC signaling, offering a targeted strategy to reduce airway obstruction.^23^ Collectively, these findings position EGFR inhibitors as promising candidates for modulating both local and systemic pathologies in COPD.

As the predominant HSP90 isoform, HSP90AA1 serves as a critical molecular chaperone in pulmonary pathophysiology by modulating the stability and functional maturation of diverse client proteins essential for cellular homeostasis.^24^ Notably, airway goblet cell metaplasia, a pathognomonic feature of COPD-associated pulmonary dysfunction,^25^ is maintained through HSP90-dependent mechanisms, with preclinical studies demonstrating that pharmacological HSP90 inhibition both reverses established metaplasia and attenuates disease progression.^26^ Importantly, HSP90 overexpression in COPD patients receiving formoterol/corticosteroid therapy correlates with glucocorticoid receptor (GR) dysfunction and impaired transcriptional activity, providing a mechanistic basis for the limited efficacy of inhaled corticosteroids in COPD management.^27^ Furthermore, HSP90-mediated downregulation of Toll-like receptor 4 (TLR4) exacerbates neutrophilic airway inflammation while simultaneously impairing glucocorticoid responsiveness, highlighting its dual role in perpetuating immune dysregulation and treatment resistance in COPD.^28^ Collectively, these findings establish HSP90AA1 as a central regulator of COPD pathogenesis, influencing goblet cell metaplasia, steroid resistance, and chronic inflammation, thereby representing a promising therapeutic target for modulating disease progression and restoring treatment efficacy. Although HSP90AA1 is a regulatory factor in the pathogenesis of COPD, its potential interaction with PFOS remains largely unexplored. Our research findings suggest that HSP90AA1 may be a key mediator in PFOS-induced COPD, although the exact mechanism requires further investigation.

Proto-oncogene tyrosine-protein kinase (SRC) has been extensively documented to regulate cellular proliferative responses elicited by growth factors, contractile agonists, and inflammatory mediators.^29^ Studies have shown that SRC promotes airway smooth muscle (ASM) growth and movement in COPD by responding to growth factors and inflammation, leading to airway thickening and worsening breathing problems.^30^ SRC demonstrates robust activation upon cigarette smoke exposure in both airway epithelial cells and alveolar macrophages. This activation, in conjunction with IL-17-driven inflammatory cascades and enhanced macrophage infiltration, synergistically potentiates pathogenic airway remodeling and accelerates parenchymal destruction characteristic of emphysema.^31^

Experimental evidence demonstrates that PFOS induces dose-dependent downregulation of estrogen receptor alpha (ESR1) expression in the testes of adult murine models.^32^ Mechanistically, PFOS dysregulates both ESR1 protein expression and downstream ERK1/2 signaling in mouse spermatocytes, where ESR1 activation has been shown to exert protective effects against PFOS-induced growth inhibition and apoptosis.^33^ Notably, this endocrine disruption extends beyond reproductive tissues, as emerging evidence indicates PFOS functions as a functional antagonist of ESR1 signaling in the central nervous system. Specifically, PFOS suppresses ESR1-dependent activation of kisspeptin neurons in the hypothalamic anteroventral periventricular (AVPV) nucleus, thereby disrupting pulsatile GnRH release and impairing ovarian cyclicity in female murine models.^34^ ESR1, a nuclear receptor protein, is closely associated with the regulation of inflammatory responses. Research has demonstrated that estrogen receptor alpha (ESR1) levels in the bronchial epithelial cells of COPD patients are markedly reduced compared to healthy individuals,^35^ likely due to the suppression of ESR1 gene transcription or protein synthesis by inflammatory factors in patients. In another study using a mouse model of acute lung inflammation induced by intratracheal lipopolysaccharide (LPS) administration, pretreatment with estrogen (17β-estradiol) significantly reduced the secretion of inflammatory factors and the infiltration of inflammatory cells, while restoring ESR1 expression to near-normal levels. This suggests that estrogen exerts anti-inflammatory effects through the activation of ESR1.^36^ Therefore, activating ESR1 may help suppress the production of inflammatory factors, thereby alleviating inflammation and lung tissue damage in COPD patients.

In network toxicology studies, enrichment analysis provides a systematic and comprehensive approach to elucidate the biological functions of targets and their associated signaling pathways.^37^ Our findings indicate that the PI3K-Akt, Ras, estrogen, MAPK, and IL-17 signaling pathways are the primary molecular mechanisms involved, which collectively contribute to the pathogenesis and progression of COPD through distinct molecular regulatory networks.

Estrogen signaling pathways play a pleiotropic regulatory role in maintaining organismal homeostasis and organ function. Animal studies demonstrate that chronic low-dose PFOS exposure suppresses the expression of key estrogen synthesis enzymes by downregulating H3K14 acetylation in the StAR promoter region, ultimately impairing follicular development.^38^ Computational analyses, including molecular docking and dynamics simulations, confirm that PFOS competitively binds to the ligand-binding domain of estrogen receptor α (ERα), sterically hindering endogenous estrogen-receptor interactions.^39^ In ovariectomy-induced estrogen-deficient mice, exogenous estrogen supplementation markedly improved pulmonary function, evidenced by a 15%–20% increase in FEV1/FVC ratio and a 25% reduction in alveolar structural damage. Moreover, estrogen mitigates oxidative damage in COPD models via Nrf2/HO-1 pathway activation, enhancing key antioxidant defenses including superoxide dismutase (SOD) and glutathione (GSH) activity.^40^ Notably, estrogen intervention in chemically induced COPD animal models consistently demonstrated pulmonary protective effects, reinforcing its therapeutic potential and identifying critical clinical targets.^41^ Paradoxically, rising COPD prevalence in females correlates with estrogen’s dual role—exacerbating disease progression via mucus hypersecretion and pro-inflammatory mechanisms.^42^^,^^43^^,^^44^ Estrogen exhibits a complex dual role in inflammation regulation, with context-dependent pro- and anti-inflammatory effects. This apparent paradox may stem from multiple factors: premenopausal women likely benefit from estrogen receptor beta (ERβ)-dominated pulmonary protection, while postmenopausal estrogen decline shifts the balance toward ERα predominance, potentially driving NF-κB activation and Nrf2 suppression linked to increased COPD susceptibility.^45^ However, this mechanism requires further validation. Therefore, we speculate that the pro-/anti-inflammatory balance of estrogen and its specific effects on pulmonary diseases may vary significantly depending on estrogen levels, individual variations, disease stages, and other factors. Further experimental investigations, clinical observations, and multifactorial integrative analyses are required to systematically elucidate the precise roles and molecular mechanisms of estrogen receptors in respiratory diseases.

The PI3K-Akt signaling pathway serves as a critical intracellular regulatory hub, modulating essential physiological processes including cell survival, proliferation, migration, and energy metabolism.^46^^,^^47^ Existing studies demonstrate that PFOS activates the PI3K-Akt pathway, enhancing colorectal cancer cell migration, accelerating hepatocellular carcinoma progression, and impairing tight junction function in brain endothelial cells.^48^^,^^49^^,^^50^ In a COPD mouse model, activation of the PI3K/Akt pathway promotes M2 polarization of alveolar macrophages,^51^ driving pro-inflammatory cytokines (TNF-α, IL-1β, and IL-6), thereby exacerbating inflammation and impairing tissue repair.^52^^,^^53^^,^^54^ Further linking PI3K-Akt to COPD pathogenesis, Histone Deacetylase (HDAC), a key glucocorticoid signaling effector, shows reduced activity that is strongly related to glucocorticoid resistance in COPD patients.^55^ Animal studies demonstrate significantly reduced HDAC bioactivity in COPD model mice, while genetic blockade of PI3K signaling restores enzymatic activity.^56^ These findings suggest that PI3K pathway inhibitors can effectively rescue HDAC dysfunction under oxidative-stress-induced glucocorticoid resistance. This regulatory mechanism not only restores the anti-inflammatory effects of glucocorticoids but may also attenuate COPD progression through this pathway. Moreover, traditional herbal medicine targeting the PI3K-Akt pathway significantly suppresses inflammatory cytokine expression in COPD mice, further validating its pivotal role in airway inflammation regulation.^57^^,^^58^

The Ras signaling pathway serves as a central regulatory mechanism for cellular responses to environmental stressors (e.g., PFOS exposure),^59^ mediating disease pathogenesis through control of proliferation, differentiation, and survival. Its dysregulation is strongly implicated in pulmonary disorders including COPD.^60^ In PFOS-exposed animal models, the Ras pathway exhibits dose-dependent activation, characterized by significant upregulation of the Rap1b/Kras-BRaf-MEK-ERK axis with elevated expression of key genes (Rap1b, Kras, and BRaf). This cascade stimulates pro-inflammatory cytokine release (IL-1β and TNF-α), ultimately inducing pulmonary tissue damage.^61^ Notably, COPD model mice demonstrate aberrant RAS protein activation and downstream signaling dysregulation, which exacerbates both inflammatory responses and pulmonary fibrosis. Targeted intervention at the RAS GTP-GDP binding domain effectively suppresses its activity and blocks downstream transduction, significantly improving both lung function parameters and histopathological features.^62^

As a crucial member of the MAPK family, p38 MAPK plays a pivotal role in cellular stress responses and inflammation regulation.^63^ Studies reveal that PFOS exposure induces p38 MAPK phosphorylation, thereby activating this signaling pathway. Such activation may ultimately disrupt cellular physiological functions by altering downstream transcription factor regulation patterns.^64^^,^^65^ In COPD patients, p38 MAPK signaling is activated in multiple pulmonary cell types and mediates distinct pathological effects. Specifically, cigarette smoke induces p38 phosphorylation in bronchial epithelial cells, promoting IL-8 secretion; alveolar macrophages exhibit enhanced p38 activity that drives excessive production of TNF-α and IL-6; while in lung fibroblasts, p38 activation contributes to airway remodeling processes. This cell-type-specific p38 activation collectively exacerbates COPD progression through coordinated inflammatory and fibrotic responses.^66^^,^^67^^,^^68^ Sputum analysis in COPD patients reveals significantly elevated p38 MAPK activity that positively correlates with disease severity. Furthermore, studies identified a strong correlation between sputum neutrophil percentage and p38 activity, while pulmonary function tests revealed a significant inverse relationship between FEV1% and p38 activation status.^69^ Notably, pharmacological inhibition of p38 MAPK significantly reduces inflammatory cytokine levels, providing both experimental validation and clinical potential for targeted COPD therapies.^70^

IL-17 serves as a pivotal inflammatory mediator in multiple pathophysiological processes. An *in vitro* human alveolar epithelial cell model demonstrated that PFOS exposure dose-dependently upregulates IL-17A/F mRNA and protein expression via STAT3 pathway activation, thereby triggering inflammatory responses.^71^ Immunohistochemical analyses revealed marked upregulation of IL-17A, RANKL, and RANK in COPD lungs, particularly within lymphoid follicles.^72^^,^^73^ Complementary *in vitro* studies demonstrated IL-17A-mediated induction of RANKL in B cells and concurrent upregulation of RANK/CXCL13 in dendritic cells, mechanistically linking IL-17A to lymphoid neogenesis via RANKL/RANK signaling. These ectopic lymphoid structures function as “chronic inflammation factories” in COPD, where the IL-17/RANKL-CXCL13 axis perpetuates pathological B cell/T cell/dendritic cell activation, driving persistent inflammation, accelerated tissue damage, and disease progression.^74^ Furthermore, IL-17 activates NF-κB signaling to promote inflammatory cell infiltration, airway hyperresponsiveness, and tissue remodeling. Anti-IL-17 therapy significantly attenuates these pathological alterations.^75^ Electroacupuncture intervention in COPD rat models demonstrates that suppressing IL-17/IL-17R expression and downstream p38/ERK/JNK MAPK signaling activation reduces pro-inflammatory cytokine release and ameliorates oxidative stress, thereby effectively alleviating airway inflammation and pulmonary tissue damage in COPD.^76^ In summary, the toxicity mechanisms of PFOS may involve multi-level signaling dysregulation, affecting inflammatory responses, oxidative stress, and tissue repair, ultimately contributing to the development and progression of COPD.

This study employed an integrated strategy combining network toxicology and molecular docking analysis to systematically elucidate the potential mechanisms of PFOS in COPD. Through rigorous screening, 158 shared targets between PFOS and COPD were identified, among which EGFR, ESR1, GRB2, HSP90AA1, and SRC were confirmed as core targets mediating PFOS-induced COPD. Molecular docking analysis further validated the high binding affinity between PFOS and these targets, supporting the reliability of their interactions. Additionally, significant enrichment of signaling pathways, including PI3K-Akt, Ras, estrogen, MAPK, and IL-17, was observed, suggesting that PFOS may contribute to COPD pathogenesis by disrupting the biological functions of these pathways. These results offer essential theoretical understanding regarding the influence of PFOS on the progression of COPD and its associated environmental health risks.

### Limitations of the study

Combining network toxicology with molecular docking utilizes progress in bioinformatics, genomics, and big data analytics to investigate the mechanisms behind the interactions of various environmental pollutants. This approach significantly enhances the efficiency of ecotoxicological research. However, our study still has certain limitations.

This study is inherently constrained by its exclusive reliance on computational approaches. While molecular docking and network toxicology provide valuable mechanistic hypotheses, the absence of experimental validation (e.g., SPR/ITC for binding affinity confirmation or cellular models for pathway verification) limits confidence in the biological relevance of predicted PFOS-protein interactions and COPD-related pathway perturbations. Computational scores cannot fully replicate dynamic physiological conditions, potentially overestimating binding stability or overlooking post-translational regulatory effects. Additionally, the discussion of research results remain speculative without evidence from disease models or clinical cohorts. Due to current resource limitations, these verifications cannot be implemented temporarily. However, we are actively pursuing collaborations to prioritize experimental confirmation of key targets (e.g., biophysical assays and *in vitro* exposure studies) in future work. Our findings should thus be interpreted as hypothesis-generating insights requiring empirical substantiation.

This work focused solely on PFOS and did not evaluate its metabolic derivatives (e.g., PFOA), which constitutes an important limitation. Although PFOS itself was prioritized for mechanistic analysis, its metabolites may exhibit divergent toxicological profiles or tissue-specific effects that could influence COPD progression through uncharacterized pathways. Our network toxicology framework and molecular docking models did not account for potential bioactivation or detoxification processes that might alter PFOS metabolism *in vivo*. Additionally, the exclusion of metabolite-protein interaction analyses limits our ability to assess confounding effects between PFOS and its derivatives. For example, PFOS metabolites (e.g., PFOA) may exhibit (1) synergistic toxicity with parent PFOS by co-activating shared pathways, (2) competitive inhibition of PFOS-binding targets, or (3) additive effects on oxidative stress through independent mechanisms. While this simplification allowed focused hypothesis generation, future investigations should integrate metabolite profiling to comprehensively evaluate environmental PFOS exposure risks.

## Resource availability

### Lead contact

Requests for further information and resources should be directed to and will be fulfilled by the lead contact, Zhenyan Huang (huangzhenyan@163.com).

### Materials availability

This study did not generate new unique reagents.

### Data and code availability

• The data related to GSEA analysis reported in this paper can be accessed from the Gene Expression Omnibus (GEO: https://www.ncbi.nlm.nih.gov/geo/). The structural data for the molecular docking of PFOS-bound targets are available in the Protein DataBank (PDB) under the following accession codes: EGFR (1ivo), ESR1 (1a52), GRB2 (1bm2), HSP90AA1 (1byq), and SRC (1a07). The authors confirm that all data underlying the findings are fully available without restriction. All relevant data are within the paper.
• This paper does not report original code.
• Any additional information required to reanalyze the data reported in this paper is available from the lead contact upon request.

## Acknowledgments

The authors would like to thank all researchers who shared their data publicly and made this project possible. This work was supported by the Medical Research Project of Zhongshan City (2024J211), Joint Research Project on Immunology by Professor Lu Chuanjian’s Qihuang Scholar Studio at Zhongshan Hospital of Traditional Chinese Medicine, No: 1, and Famous Traditional Chinese Medicine Inheritance Studio Construction Project of Zhongshan City.

## Author contributions

C.P. and J.W., writing—original draft, visualization, methodology, and data curation. W.C., writing—original draft and data curation. F.L., visualization and software. Z.H. and Y.H., writing—review & editing and conceptualization.

## Declaration of interests

The authors declare no competing interests.

## STAR★Methods

### Key resources table

**Table:** 

REAGENT or RESOURCE	SOURCE	IDENTIFIER
**Deposited data**		

Raw and analyzed data	This paper	GEO: GSE54837; GSE76925; GSE130928
B-RAF RBD (apo) structure for molecular docking	Zhang, X. F. et al.	PDB: 1IVO,1A52,1BM2,1BYQ,1A07
3D structure of PFOS	Jane, L. E. L. et al.	PubChem ClD:74483

**Software and algorithms**		

PubChem database	Jane, L. E. L. et al.	https://pubchem.ncbi.nlm.nih.gov/
ProTox-II	Marwick, J. A. et al.	https://tox.charite.de/protox3/
ADMETlab	Xie, Y. et al.	https://admetlab3.scbdd.com/
STITCH	Peng, J., He, J. et al.	https://ngdc.cncb.ac.cn/databasecommons/database/id/208
SwissTargetPrediction	Zhang, M. et al.	http://www.swisstargetprediction.ch/
PharmMapper database	Zhao, X. et al.	https://lilab-ecust.cn/pharmmapper/index.html
UniProt database	Shi, X. & Zhou, B. et al.	https://www.uniprot.org/id-mapping/
GeneCards	Renda, T. et al.	https://www.genecards.org/
OMIM	Pelaia, C. et al.	https://www.omim.org/
TTD	Huang, C. et al.	https://db.idrblab.net/ttd/
Venny 2.1.0	Santos, A.P. et al.	https://bioinfogp.cnb.csic.es/tools/venny/
STRING 12.0 database	Gaffey, K. et al.	https://string-db.org
SangerBox platform	Eustace, A. et al.	http://sangerbox.com/
GEO database	Barrett, T. et al.	https://www.ncbi.nlm.nih.gov/geo/
RCSB Protein Data Bank	Zhang, X. F. et al.	http://www.rcsb.org
CB-Dock2 online tool	Camargo, L. D. N. et al.	https://cadd.labshare.cn/cb-dock2/
Cytoscape 3.10.1 software	Chen, J. et al.	https://cytoscape.org/

### Experimental model and study participant details

#### Study design and patients

The public GEO datasets GSE54837, GSE76925, and GSE130928 were used in this study. These datasets include whole blood samples from COPD patients (136 cases/84 controls), lung tissue samples from COPD patients (111 cases/40 controls), and alveolar macrophages from bronchoalveolar lavage in COPD patients (22 cases/42 controls), respectively.

#### Methods

##### Preliminary network-based analysis of PFOS toxicity

The ProTox platform integrates molecular similarity algorithms with advanced machine-learning models to predict 61 distinct toxicity endpoints. These include acute toxicity, organ-specific toxicity, clinical toxicity manifestations, molecular-initiating events, adverse outcome pathways, along with various other toxicological endpoints and potential off-target toxicity profiles.^77^ The ADMETlab platform offers comprehensive pharmacokinetic profiling of candidate compounds, evaluating key ADMET (absorption, distribution, metabolism, excretion, and toxicity) properties. Furthermore, the platform provides extensive toxicological assessments, including detailed analyses of compound toxicity profile.^78^

We retrieved the canonical SMILES representation of PFOS by querying “Perfluorooctane sulfonate” in the PubChem database (https://pubchem.ncbi.nlm.nih.gov/).^79^^,^^80^ The structural information of PFOS was then imported into the computational toxicology prediction platforms ProTox-II (https://tox.charite.de/protox3/)^77^ and ADMETlab (https://admetlab3.scbdd.com/).^78^^,^^81^ By integrating the predictive results from both platforms, a preliminary analysis and toxicity assessment of PFOS were conducted.

##### Collection of PFOS-related targets

The STITCH database systematically integrates experimentally validated and computationally predicted compound-protein interactions, constructing a global network encompassing both protein-protein and protein-chemical interactions through the synthesis of five primary data types: genomic context predictions, high-throughput experiments, co-expression patterns, text-mining results, and curated database knowledge.^82^ As a complementary resource, SwissTargetPrediction employs advanced similarity-based algorithms and reverse pharmacophore screening to predict small molecule-protein target interactions, drawing from its extensive collection of 376,342 bioactive compounds with experimentally confirmed activity against 3,068 distinct targets.^83^ For pharmacophore-based target identification, the PharmMapper Server provides an open-access platform featuring 23,236 proteins with 16,159 druggable and 51,431 ligandable pharmacophore models, facilitating target prediction for drugs, natural products, and critical compounds.^84^

It is important to emphasize that PFOS, the primary focus of this investigation, represents an environmental contaminant rather than a pharmaceutical compound. The application of predictive tools primarily optimized for drug discovery to identify PFOS-associated proteins may introduce systematic prediction bias. This constitutes both a limitation of the present study and a broader methodological challenge in contemporary environmental toxicology research.

We systematically screened potential targets of PFOS using a multi-database integration strategy. Initially, the SMILES molecular structure of PFOS was submitted to both the STITCH (https://ngdc.cncb.ac.cn/databasecommons/database/id/208) and Swiss Target Prediction databases (http://www.swisstargetprediction.ch/) for target prediction.^85^ To comprehensively explore potential targets, the SMILES structure was converted into SDF format and further analyzed using the PharmMapper database (https://lilab-ecust.cn/pharmmapper/index.html) for supplementary target identification. Subsequently, the prediction results from these three databases were integrated, and duplicate entries were removed to obtain a non-redundant target set. To ensure the standardization and accuracy of target nomenclature, UniProt IDs were extracted for all targets, and the UniProt database (https://www.uniprot.org/id-mapping/)^86^ was utilized for normalization. Through this systematic screening process, a comprehensive target database for PFOS was successfully established, providing a robust data foundation for subsequent network toxicology research.

##### Screening of COPD-associated molecular targets

GeneCards serves as a premier integrated gene compendium, aggregating genomic, proteomic, transcriptomic, and disease-associated annotations from >150 sources into unified gene profiles.^87^ OMIM (Online Mendelian Inheritance in Man) represents the gold-standard repository for curated genotype-phenotype relationships, providing expert-evaluated evidence linking genomic variants to heritable disorders, with NIH-maintained clinical and molecular annotations supporting genomic medicine applications.^88^^,^^89^ Complementing these resources, TTD (Therapeutic Target Database) offers a systematically curated knowledgebase of validated therapeutic targets, their pharmacological modulators, mechanisms, and developmental statuses, enabling target-disease-drug connectivity analysis for discovery research.^90^

By integrating data resources from GeneCards (https://www.genecards.org/), OMIM (https://www.omim.org/), and TTD databases (https://db.idrblab.net/ttd/), we conducted a comprehensive search using “COPD” as the key term to identify and screen potential targets associated with COPD. Subsequently, a cross-comparison analysis was performed to map PFOS-related molecular targets against the COPD target set, enabling the identification of shared targets between the two. To visually represent the analytical findings, Venn diagram visualization was employed to systematically illustrate the interaction network and overlapping relationships between PFOS and COPD at the molecular target level.

##### Construction of protein-protein interaction (PPI) network and screening of core targets

STRING is a premier database of known and predicted protein-protein interactions, integrating experimental evidence, computational predictions, and curated pathway knowledge to construct comprehensive interaction networks with confidence scoring. This widely-used resource enables systematic analysis of functional protein associations through physical and functional linkages across >14,000 organisms.^91^ The shared target gene set between PFOS and COPD was imported into the STRING 12.0 database (https://string-db.org), with the species limited to “Homo sapiens” and an interaction confidence score threshold set to >0.4 (medium confidence) to construct a protein-protein interaction (PPI) network.^92^ The confidence score cutoff of >0.4 was selected based on STRING's benchmarking studies demonstrating this threshold optimally balances sensitivity (74% recall of known interactions) and precision (56% of predicted interactions being experimentally verified), while maintaining sufficient network connectivity for comprehensive pathway analysis.^93^ Subsequently, Cytoscape 3.10.1 software (https://cytoscape.org/)^94^ was utilized for network visualization and topological analysis. Core targets were determined through the analysis of topological parameters, including degree centrality and betweenness centrality. Based on the network topology characteristics, the molecular toxicity mechanisms of PFOS and its potential association with COPD were systematically elucidated.

##### Functional annotation and pathway enrichment analysis of target proteins

We utilized the SangerBox platform (http://sangerbox.com/)^95^ to perform Gene Ontology (GO) and Kyoto Encyclopedia of Genes and Genomes (KEGG) pathway enrichment analyses, aiming to comprehensively elucidate the functional annotations and pathway associations of potential target genes. The GO analysis systematically revealed the functional characteristics of these genes across three domains: biological processes, molecular functions, and cellular components. Meanwhile, the KEGG analysis further identified their enrichment in key signaling pathways and metabolic networks. These results offer essential molecular perspectives on the toxicological mechanisms of PFOS and its potential associations with diseases such as COPD.

##### Gene set enrichment analysis (GSEA)

Subsequently, we conducted a systematic validation of five KEGG signaling pathways - PI3K-Akt, Ras, Estrogen, MAPK, and IL-17 - through Gene Set Enrichment Analysis (GSEA) to assess their potential roles in PFOS-induced COPD.^96^ The study analyzed three independent COPD-related transcriptomic datasets obtained from the Gene Expression Omnibus (GEO) database (https://www.ncbi.nlm.nih.gov/geo/): GSE54837 (whole blood samples from COPD patients, n=136 cases/84 controls), GSE76925 (lung tissue samples from COPD patients, n=111 cases/40 controls), and GSE130928 (alveolar macrophages from bronchoalveolar lavage in COPD patients, n=22 cases/42 controls). The GEO dataset samples were analyzed using a case-control design, with the experimental group comprising patients meeting COPD diagnostic criteria and the control group consisting of smokers without COPD diagnosis, as shown in Table 5. Considering the potential biological heterogeneity across different sample sources, we performed GSEA independent analyses for each dataset using the SangerBox platform, employing weighted enrichment statistics and signal-to-noise ratio ranking metrics, with stringent significance thresholds (|NES| > 1, nominal P-value < 0.05, FDR < 0.25). This multi-dataset analytical strategy based on diverse tissue sources, not only enables validation of pathway consistency across different biological samples but also identifies tissue-specific regulatory patterns of these pathways, thereby providing multi-dimensional bioinformatics evidence for elucidating the molecular mechanisms linking PFOS exposure to COPD pathogenesis.

**Table 5:** Details of the datasets used in GSEA

GEO accession	GEO platform	Test group vs. control group	Source	Organism
GSE54837	GPL570	COPD patients (*N* = 136) vs. smoker controls (*N* = 84)	Whole blood	Homo sapiens
GSE76925	GPL10558	COPD patients (*N* = 111) vs. smoker controls (*N* = 40)	Lung tissue	Homo sapiens
GSE130928	GPL570	COPD patients (*N* = 22) vs. smoker controls (*N* = 42)	Alveolar macrophages from bronchoalveolar lavage	Homo sapiens

The GEO dataset samples were analyzed using a case-control design, with the experimental group comprising patients meeting COPD diagnostic criteria and the control group consisting of smokers without COPD diagnosis. The analysis comprised 435 total subjects (253 COPD cases vs. 182 smoker controls).

##### Validation of molecular interactions through docking studies

CB-Dock2 is an advanced protein-ligand blind docking server that integrates structure-based cavity detection with template-based molecular docking, achieving an 85.9% success rate in binding pose prediction by leveraging a curated database of 214,506 protein-ligand complexes from the Biological Ligand-Protein interaction database (BioLip).^97^ This freely accessible web tool combines automated cavity detection, dual docking pipelines, and interactive visualization features to facilitate efficient drug discovery and protein function annotation.

To validate the interactions between PFOS and potential targets, molecular docking experiments were conducted using the CB-Dock2 online docking tool (https://cadd.labshare.cn/cb-dock2/) in conjunction with the RCSB Protein Data Bank (http://www.rcsb.org).^98^ Initially, the three-dimensional structures of target proteins were retrieved from the RCSB Protein Data Bank and prepared as receptor molecules for subsequent analyses. Subsequently, the molecular structure of PFOS was employed as the ligand, and docking simulations were performed using CB-Dock2 to predict its binding modes and affinity with the target proteins. In CB-Dock2, the molecular docking parameters were configured as follows: Binding sites were detected through dual approaches, including structure-based prediction via protein surface curvature analysis (CurPocket algorithm) and template-based matching using homologous complexes from the BioLip database (ligand FP2 similarity ≥0.4). Cavities sharing >50% binding residue overlap were automatically merged. Conformation generation employed parallel pipelines: structure-guided docking (AutoDock Vina with default parameters) and template-guided docking (FitDock hierarchical alignment with 7-layer atom matching and BFGS optimization with step size ≤0.5 Å). All output conformations were ranked according to the AutoDock Vina scoring function, with only the top-scoring conformation retained per cavity (docking success criterion: RMSD <2.0 Å). For the obtained results, we selected the three most favorable binding positions (ranked in ascending order of binding energy) for visualization, representing the ligand-receptor interactions with optimal energy.

### Method details

#### Toxicity assessment of PFOS

We retrieved the canonical SMILES representation of PFOS by querying “Perfluorooctane sulfonate” in the PubChem database (https://pubchem.ncbi.nlm.nih.gov/).^80^ Subsequently, the SMILES structural information of PFOS was imported into the computational toxicology prediction platforms ProTox-II (https://tox.charite.de/protox3/)^77^ and ADMETlab (https://admetlab3.scbdd.com/).^78^ By integrating the prediction results from these two platforms, it was revealed that the active toxicity endpoints of PFOS are closely associated with respiratory toxicity.

#### Collection of PFOS targets

The SMILES molecular structure of PFOS was submitted to the STITCH (https://ngdc.cncb.ac.cn/databasecommons/database/id/208)^82^ and SwissTargetPrediction (http://www.swisstargetprediction.ch/)^83^ databases for target prediction. Additionally, the SMILES structure was converted to SDF format and imported into the PharmMapper database (https://lilab-ecust.cn/pharmmapper/index.html)^84^ for further analysis. After integrating the prediction results from these three databases and removing redundant entries, 283 potential PFOS-related targets were identified. These targets were then standardized using the UniProt database (https://www.uniprot.org/id-mapping/)^86^ for consistent nomenclature.

#### Screening of COPD-associated molecular targets

Using “COPD” as the keyword, a comprehensive search was conducted across the GeneCards (https://www.genecards.org/),^87^ OMIM (https://www.omim.org/),^89^ and TTD (https://db.idrblab.net/ttd/)^90^ databases, identifying 2,637 high-confidence COPD-related targets. Subsequently, Venny 2.1.0 (https://bioinfogp.cnb.csic.es/tools/venny/)^99^ was employed to perform an intersection analysis between the two target sets, revealing 158 overlapping targets linking PFOS and COPD.

#### Construction of protein-protein interaction (PPI) network and screening of core targets

The 158 overlapping targets between PFOS and COPD were imported into the STRING 12.0 database (https://string-db.org),^91^ with the species restricted to *Homo sapiens* and the minimum interaction confidence score set to >0.4 (medium confidence). A protein-protein interaction (PPI) network and TSV file were generated, comprising 158 nodes and 1,989 edges. The average node degree of the network was 25.2, indicating extensive interactions among the targets. Subsequently, the TSV file was opened using Cytoscape 3.10.1 software for network visualization and topological analysis, producing an intuitive structural diagram of the PPI network. Based on topological parameters such as Degree Centrality, Betweenness Centrality, and Closeness Centrality, five core targets were ultimately identified: EGFR, ESR1, GRB2, HSP90AA1, and SRC.

#### Functional annotation and pathway enrichment analysis of target proteins

The 158 overlapping targets between PFOS and COPD were uploaded to the SangerBox platform (http://sangerbox.com/)^95^ for Gene Ontology (GO) analysis, with thresholds set at FDR < 0.1 and p-value < 0.05. A total of 3,547 significantly enriched GO terms were identified, systematically categorized into 3,059 biological processes (BP), 140 cellular components (CC), and 348 molecular functions (MF). To identify the most representative GO terms, the results were sorted by p-value, and the top 10 entries with the lowest p-values were selected from each category (BP, CC, and MF). Additionally, KEGG pathway enrichment analysis was performed on the 158 potential targets using the SangerBox platform. The analysis identified 157 significantly enriched signaling pathways, and the top 20 most significant KEGG pathways were visualized for further interpretation.

#### Gene set enrichment analysis (GSEA)

We selected three COPD-related transcriptomic datasets from the GEO database (https://www.ncbi.nlm.nih.gov/geo/)^100^ based on the following criteria: (1) sufficient sample size (>20 per group); (2) inclusion of both case and control samples; and (3) direct relevance to chronic obstructive pulmonary disease (COPD). The specific datasets included: GSE54837 (whole blood samples from COPD patients, n=136 cases/84 controls), GSE76925 (lung tissue samples from COPD patients, n=111 cases/40 controls), and GSE130928 (alveolar macrophages from bronchoalveolar lavage in COPD patients, n=22 cases/42 controls). After importing the raw expression profile data (TXT format) of these three datasets into the SangerBox analysis platform, we first extracted KEGG pathway annotation information. We then selected five target pathways for validation from these annotations and imported the corresponding pathway datasets into the platform's custom database module, ultimately completing subsequent bioinformatics analyses.

#### Validation of molecular interactions through docking studies

The five core target proteins—EGFR (PDB: 1ivo), ESR1 (PDB: 1a52), GRB2 (PDB: 1bm2), HSP90AA1 (PDB: 1byq), and SRC (PDB: 1a07)—were retrieved from the RCSB Protein Data Bank (http://www.rcsb.org)^98^ in PDB format and subsequently converted into PDBQT files for use as receptor molecules in further analyses. The 3D conformer SDF structure file of PFOS, previously obtained from the PubChem database, was employed as the ligand. Molecular docking simulations were performed using the CB-Dock2 online tool (https://cadd.labshare.cn/cb-dock2/).^97^ The PDBQT files (receptors) and the SDF file (ligand) were uploaded to CB-Dock2, with the “Number of cavities for docking” parameter set to 5 (default value). The docking simulations were then executed, and the resulting binding poses were visualized.

### Quantification and statistical analysis

Statistical analyses were performed using established bioinformatics methods with stringent significance thresholds. Protein-protein interaction networks were constructed in STRING database (v12.0) applying Fisher's exact test to determine interaction significance, with a confidence score cutoff >0.4 for network inclusion. Functional enrichment analyses employed hypergeometric testing with Benjamini-Hochberg multiple testing correction, considering GO terms significant at FDR <0.1 and p <0.05 (top 10 terms shown per category) and KEGG pathways significant at p <0.05 (top 20 pathways shown). GSEA was performed on the SangerBox platform using weighted enrichment statistics for GEO datasets (GSE54837, GSE76925, GSE130928). Through 1000 gene-set permutations, the significance thresholds were set as: normalized enrichment score (|NES|) >1, nominal P-value (∗P<0.05, ∗∗P<0.01), and false discovery rate (FDR q) <0.25. Molecular docking results represent mean binding energies ± SD from three independent CB-Dock2 runs. All statistical tests were two-tailed with p <0.05 considered significant, and data visualization was performed using Cytoscape 3.10.1 for network analysis and standard bioinformatics plotting tools for enrichment results.

## Supplemental information

Document S1. The three TXT format datasets obtained from the GEO database, related to Figure 6 and Table 4

Document S2. The PDB (Protein DataBank) format files of the five core target proteins used as receptor molecules, and the 3D conformer SDF (Structure Data File) structure files of PFOS, related to Figure 7 and Table 5

Table S1. Potential molecular targets of PFOS, related to Figure 2

Table S2. COPD-associated molecular targets, related to Figure 2

Table S3. The overlapping targets between PFOS and COPD, related to Figure 2

Table S4. GO enrichment analysis by the SangerBox platform, related to Figure 4

Table S5. KEGG signaling pathway analysis by the SangerBox platform, related to Figure 5
